# Tri‐modality therapy with i‐125 brachytherapy, external beam radiation therapy, and short‐term hormone therapy for high‐risk prostate cancer after holmium laser enucleation of the prostate

**DOI:** 10.1002/iju5.12437

**Published:** 2022-03-30

**Authors:** Makoto Nakiri, Kosuke Ueda, Naoyuki Ogasawara, Hirofumi Kurose, Keiichiro Uemura, Kiyoaki Nishihara, Koichiro Muraki, Chikayuki Hattori, Etsuyo Ogo, Tsukasa Igawa

**Affiliations:** ^1^ Departments of Urology Kurume University School of Medicine Kurume Japan; ^2^ Department of Radiology Kurume University School of Medicine Kurume Japan

**Keywords:** benign prostatic hyperplasia, brachytherapy, dysuria, holmium, prostate cancer

## Abstract

**Introduction:**

We present tri‐modality therapy with i‐125 brachytherapy for high‐risk prostate cancer after holmium laser enucleation of the prostate.

**Case presentation:**

A 75‐year‐old man had visited our hospital with complaints of dysuria. Holmium laser enucleation of the prostate was performed for benign prostatic hyperplasia. The resected histopathological prostate tissue showed malignancy (Gleason score: 3 + 3 = 6). Two years thereafter, Gleason score progressed (4 + 5 = 9) concomitantly with increased prostate‐specific antigen levels. Therefore, tri‐modality therapy, including brachytherapy, was applied. Combined androgen blockade therapy was conducted over a 9‐month period. One month after brachytherapy, external beam radiation was performed.

**Conclusion:**

Brachytherapy following transurethral prostate surgery is relatively contraindicated because of increased adverse urethral event frequency and seed placement difficulties. A tri‐modality therapy, including brachytherapy, was implemented without any major problems in this patient with high‐risk prostate cancer after holmium laser enucleation of the prostate, following which he had a favorable prognosis without recurrence for 6 years.

**Abbreviations & Acronyms**
BPH: benign prostate hyperplasia
BT: Brachytherapy
CAB: Combined androgen blockade
DVH: dose–volume histogram
GS: Gleason score
HoLEP: holmium laser enucleation of the prostate
IMRT: intensity‐modulated radiation therapy
PSA: prostate‐specific antigen

**Keynote message**

Tri‐modality therapy, including brachytherapy post‐holmium laser enucleation, was safe with no recurrence of high‐risk prostate cancer. Tri‐modality therapy, including brachytherapy, is effective for high‐risk cases. Successful tri‐modality therapy requires improved radiation seed placement skills and close coordination with radiologists.

## Background

Prostate cancer treatments include surgery, radiation therapy, and hormone therapy. BT, IMRT, and particle beam therapy are also widely used. Permanent insertion of I‐125 seeds is the standard treatment, with a biochemical non‐recurrence rate comparable to that of surgery.^1^, ^2^ Although initially used in low‐risk cases, external beam radiation can be combined with hormone therapy for better therapeutic effects in intermediate‐ and high‐risk cases.^3^, ^4^, ^5^, ^6^ We present a trimodal therapeutic approach, including BT for high‐risk prostate cancer following HoLEP, which is often contraindicated given source placement difficulty and requirement of postoperative urethral dose.

## Case presentation

### Patient: A 75‐year‐old man

Chief complaint: Dysuria with benign prostate hyperplasia (prostate weight: 177 cc).

Medical history: Cerebral infarction in 2005; on anticoagulants since.

Family history: None.

## Investigations

**Fig. 1:**
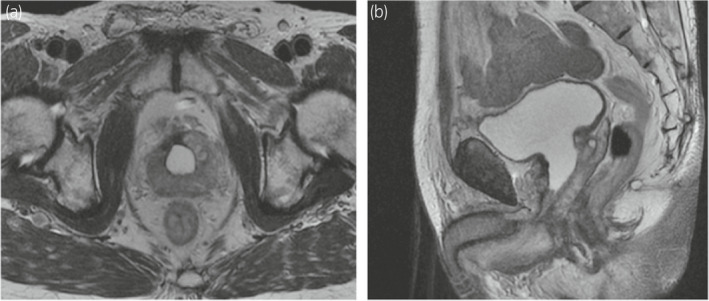
Magnetic resonance imaging prior to the start of brachytherapy––horizontal (a) and sagittal (b) views.

Magnetic resonance imaging (MRI) before permanent BT revealed cavities due to prostatic urethral HoLEP adenoma enucleation (Fig. 1a,b).

### Differential diagnosis

**Fig. 2:**
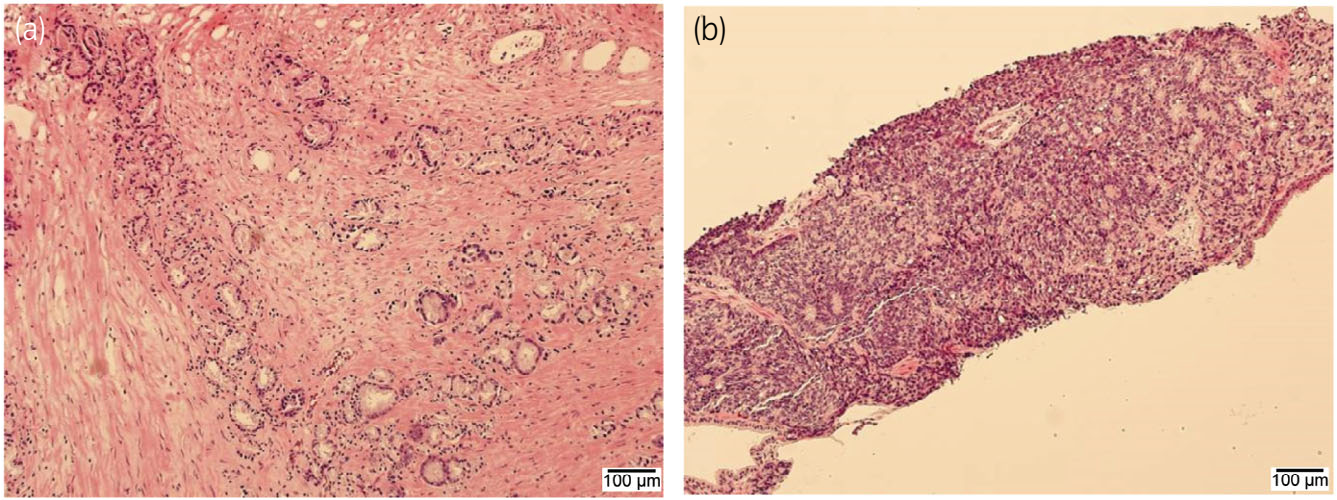
Pathological findings of holmium laser enucleation of the prostate (a) and the second prostate biopsy (b). [Colour figure can be viewed at wileyonlinelibrary.com]

The patient's PSA level was 13.7 ng/mL in 2011. Transrectal prostate needle biopsy revealed no malignancies. The enucleated tissue weighed 86 g. Pathology revealed prostate adenocarcinoma (Gleason score: 3 + 3 = 6 and cT1aN0M0). The PSA level decreased postoperatively (1.960 ng/mL). In 2013, the PSA level was elevated again (4.730 ng/mL). Repeat prostate needle biopsy revealed histological progression (Gleason score: 4 + 5 = 9) (Fig. 2a,b). Pretreatment MRI showed no significant findings, and the patient was diagnosed with cT1cN0M0 prostate cancer.

## Treatment

HoLEP was performed for BPH in 2011. Following a histopathological diagnosis of malignancy, we implemented our tri‐modality therapy protocol, including BT (Table 1).

**Table 1:** Brachytherapy protocol at our hospital

	Criteria	Treatment
Low risk	PSA <10 ng/mL and GS = 6 and ≦cT2a	Brachytherapy 145 Gy
Intermediate risk	PSA 10–20 ng/mL or GS = 7 or cT2b	Brachytherapy 110 Gy External irradiation 45 Gy
	Only one of these factors (PSA 10–20 ng/mL, GS, clinical stage) or GS = 3 + 4 + positive core <33%	Brachytherapy 145 Gy
High risk	PSA >20 ng/mL or GS ≧8 or cT2c	Brachytherapy 110 Gy External irradiation 45 Gy Hormone therapy 9 months

Abbreviations: PSA: prostate‐specific antigen, GS: Gleason score.

CAB therapy with bicalutamide and goserelin was initiated in 2014, and BT (prescribed dose: 110 Gy) was performed later. Dose distribution for preoperative planning ultrasound is depicted in Figure S1. Although difficult because of post‐HoLEP cavity formation, 67 seeds were placed during BT without complications. The urethral catheter was removed on the first posttreatment day. Three base side seeds were lost during spontaneous urination. The immediate DVH and 1‐month DVH are described in Table 2. As the base side dose was slightly insufficient, the prostate dose distribution was graded using IMRT. A total dose of 50.4 Gy was administered by adding 5.4 Gy to the 45‐Gy protocol dose. CAB was continued for 5 months after a 4‐month BT course, including pretreatment therapy.

**Table 2:** Dose–volume histogram immediately after treatment and at 1 month

Dose information		Immediately after treatment		At 1 month	
Total volume		26.25 cc		26.01 cc	
Prostate	V100%	20.10 cc	[95.37%]	24.32 cc	[93.50%]
	D90%	121.17 Gy	[110.15%]	124.95 Gy	[113.59%]
Urethra	D30%	87.11 Gy	[79.19%]	138.03 Gy	[125.48%]
Rectum	V100%	0.00 cc	[0.00%]	0.65 cc	[3.11%]

## Outcome and follow‐up

No recurrence was observed 6 years after BT. α1‐blockers were suspended for 2 months after BT with a favorable prognosis, and no obvious adverse events (including urinary disorders) were noted.

## Discussion

BT‐based approaches, including external beam radiation therapy, provide superior long‐term oncologic and functional outcomes for intermediate‐ and high‐risk prostate cancers.^4^ Several studies have found that the trimodal approach is a treatment option in high‐risk cases.^3^, ^5^, ^6^ We developed a BT protocol (Table 1) based on a previous study^7^ and administered tri‐modality therapy, including BT, for high‐risk prostate cancer.

In HoLEP, the intraprostatic gland is enucleated using a holmium laser. It can be performed safely even for relatively severe BPH.^8^ Radical prostatectomy, especially robot‐assisted radical prostatectomy, is one of the treatment options in cases with transurethral surgery. However, these cases have some technical difficulties.^8^, ^9^, ^10^, ^11^ The American Brachytherapy Society has also indicated BT as a contraindication owing to technical difficulties of seed placement.

Our patient had two risks factors: high‐risk prostate cancer and a history of HoLEP. Furthermore, he was being administered oral anticoagulants for a cerebral infarction history. The urethra and rectum were difficult to avoid with IMRT monotherapy or particle beam therapy because the prostatic urethral cavity was large. Increased urethral and rectal doses could have caused bleeding from late complications such as urethritis and proctitis. This patient also had high‐risk prostate cancer, with a volume > 10 mm (rectal side) and >5 mm (lateral lobe) observed on MRI. Therefore, tri‐modality therapy centered on BT was administered, with the possibility of reducing the urethral and rectal doses. Seed placement was difficult because they had to be placed >5 mm from the rectum after considering the urethral and rectal doses placed directly under or on the prostate capsule.

Prostate cancer of the marginal area occurs in 70% of cases^12^; therefore, the marginal placement method is performed from the initiation of BT to avoid adverse urethral events. In this case, the bladder neck‐prostate boundary was unclear on ultrasound, and the prostate margin was unclear. Nonetheless, BT was performed without major problems. However, three seeds were lost on the second postoperative day. Although the reduction in the post‐plan DVH dose after 1 month was unclear (Table 2), we consulted a radiologist considering a decrease in the base side dose.

Subsequently, the prostate dose distribution was graded with IMRT, and a total dose of 50.4 Gy was administered by adding 5.4 Gy to a 45‐Gy protocol dose. Doses can be administered locally in combination with IMRT. Furthermore, as this approach was initiated after HoLEP, complications due to physical obstructions unique to BT were not encountered (e.g., dysuria). The patient had a favorable outcome and did not require pharmacological therapy such as α1‐blockers.

Post‐plan DVH showed that the UD30 and RV100 increased relative to DVH immediately postoperatively. Because HoLEP causes intraprostatic gland hollowing, the prostate contour can be deformed when the urethral catheter is placed, and compression is applied to the rectal side (i.e., during surgery) as opposed to when no urethral catheter or rectal side‐compression is applied (i.e., post‐plan). Additionally, the positional relationship between the urethra and rectum, including the contour, could change because of prostate edema immediately after radiation source placement compared to 1 month later (i.e., post‐plan). Considering both possibilities, BT allowed a significant reduction in the urethral and rectal doses compared to IMRT monotherapy and particle beam therapy, making this development very important for patients on anticoagulants.

Careful cooperation with radiologists during surgery, pre‐plan, and post‐plan, combined with improved technical radiation source placement skills, is required to safely administer BT to patients with prostate cancer after transurethral prostate surgery.

## Author Contributions

Makoto Nakiri: Conceptualization; data curation; investigation; methodology; project administration; resources; validation; writing – original draft; writing – review and editing. Kosuke Ueda: Project administration; writing – original draft; writing – review and editing. Naoyuki Ogasawara: Conceptualization; data curation; methodology; project administration. Hirofumi Kurose: Data curation. Keiichiro Uemura: Data curation. Kiyoaki Nishihara: Data curation. Koichiro Muraki: Conceptualization; investigation; resources. Chikayuki Hattori: Methodology; resources. Etsuyo Ogo: Conceptualization; supervision. Tsukasa Igawa: Conceptualization; project administration; supervision; writing – review and editing.

## Conflict of interest

The authors declare no conflict of interest.

## Approval of the research protocol by an Institutional Review Board

Not applicable.

## Informed consent

The patient provided written informed consent for all treatment.

## Registry and the Registration No. of the study/trial

Not applicable.

## Supporting information


**Figure S1.** Dose distribution for planning ultrasound study.

## Data Availability

All data used in this study are in the manuscript, tables, and figures.
