# Supplementary figures and images for: Tri‐modality therapy with i‐125 brachytherapy, external beam radiation therapy, and short‐term hormone therapy for high‐risk prostate cancer after holmium laser enucleation of the prostate
Source: IJU Case Rep. 2022 Mar 30;5(4):223–6. doi: 10.1002/iju5.12437 (PMC9249659; doi:10.1002/iju5.12437)

**Supplementary Figure**

**
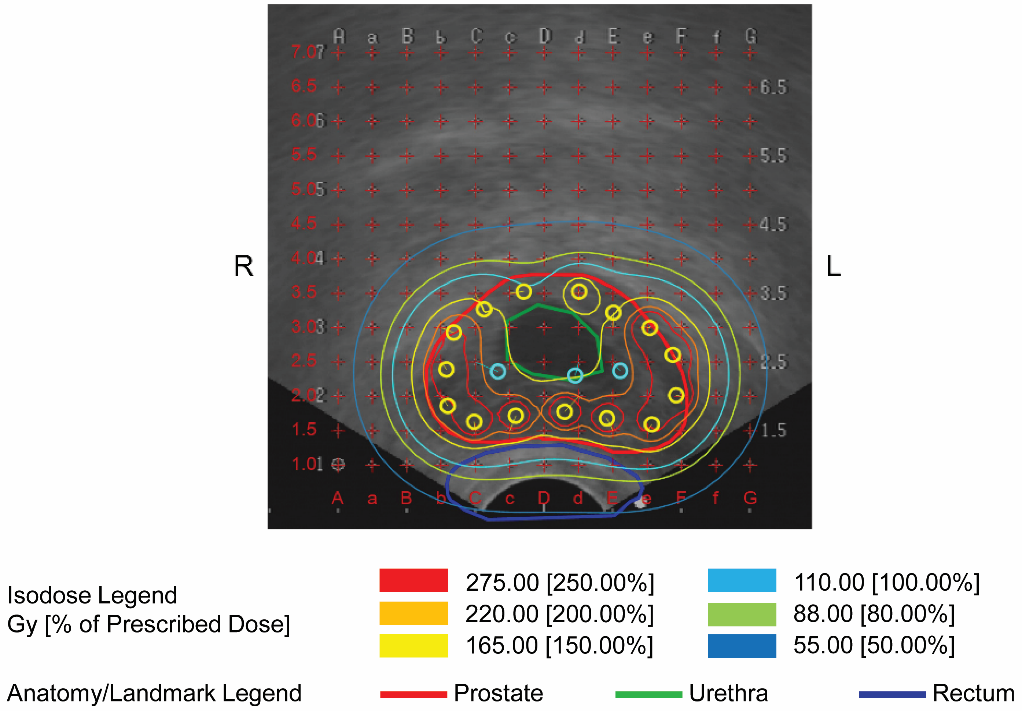
**

**Figure S1.** Dose distribution for planning ultrasound study

Supplement: Supplementary file 1 — Figure S1. Dose distribution for planning ultrasound study. [file IJU5-5-223-s001.docx]
